# Is Instillational Topical Negative Pressure Wound Therapy in Peri-Prosthetic Infections of the Breast Effective? A Pilot Study

**DOI:** 10.3390/jpm12122054

**Published:** 2022-12-13

**Authors:** Jasmin S. Gruener, Raymund E. Horch, Alexander Geierlehner, Wibke Mueller-Seubert, Aijia Cai, Andreas Arkudas, Ingo Ludolph

**Affiliations:** Laboratory for Tissue Engineering and Regenerative Medicine, Department of Plastic and Hand Surgery, University Hospital Erlangen, Friedrich-Alexander-University of Erlangen-Nürnberg (FAU), 91054 Erlangen, Germany

**Keywords:** breast infection, peri-prosthetic infection, breast implant infection, silicone prostheses, negative pressure wound therapy, instillation

## Abstract

Peri-prosthetic breast infections pose a risk of severe complications after breast implant surgery. The need to remove the breast implant, control the infection and perform additional surgical procedures are the consequences. Reimplantation of an alloplastic implant is only appropriate after an infection-free interval. In this retrospective cohort study, we investigated the effectiveness of negative pressure wound treatment with instillation and dwell time (NPWTi-d) on peri-prosthetic breast infections in combination with implant removal and antibiotic therapy. Twelve patients treated with NPWTi-d due to breast implant infection were included in the study. The bacterial burden was analyzed using wound swabs before and after NPWTi-d. Additionally, laboratory values were determined before NPWTi-d and immediately before wound closure. A total of 13 peri-prosthetic breast infections in 12 patients were treated using implant removal and NPWTi-d. In 76.9% (*n* = 10) of the cases, the patients had undergone alloplastic breast reconstruction following cancer-related mastectomy, whereas 23.1% (*n* = 3) of the patients had undergone breast augmentation for cosmetic reasons. The bacterial burden in the breast pocket decreased statistically significant after implant removal and NPWTi-d. No shift from Gram-positive to Gram-negative bacteria was observed. Inflammatory markers rapidly decreased following treatment. NPWTi-d had a positive impact on the healing process after peri-prosthetic breast infections, leading to a decrease in bacterial burden within the wounds and contributing to uneventful healing. Therefore, secondary reimplantation of breast prostheses might be positively influenced when compared to conventional implant removal and simple secondary closure. Further studies are required to conclusively establish the beneficial long-term effects of using NPWTi-d for the treatment of peri-prosthetic breast infections.

## 1. Introduction

According to the literature, peri-prosthetic breast infections are relatively rare events. Although autologous breast reconstruction has become a standard procedure, alloplastic breast augmentation and breast reconstruction with silicone implants continue to be popular procedures [1,2,3,4,5].

Peri-prosthetic breast infection (PPBI) is still a severe complication that is associated with surgical site complications and implant loss. In particular, patients undergoing tumor-associated radiation therapy of the breast before or after alloplastic breast reconstruction commonly suffer from wound-healing disorders and serious infections. Thus far, there is no consensus regarding the optimal treatment for PPBI. Standard treatment options include removal of the implant and prolonged administration of intravenous antibiotics. Subsequent issues include loss or contracture of the implant pocket, which means that the patient may need additional alloplastic or autologous breast reconstruction. Negative pressure wound therapy with instillation and dwell time (NPWTi-d) has only anecdotally been described in the context of PPBI [6].

More recent data describe the advantages of using instillational negative pressure wound therapy (NPWT) with different rinsing solutions for the treatment of acute and chronic wounds, infectious wounds and even burn injuries [7,8,9,10,11,12,13,14]. NPWTi-d with an antiseptic solution achieves a greater reduction in the number of pathogenic species within a wound compared to NPWT with no solution [15]. Furthermore, duration of hospitalization and days to wound closure in complex infected wounds can be significantly reduced with NPWTi-d compared to traditional wound care. Consequently, there can be a reduction in the cost of treating complex wounds [16,17]. However, little is known about the use of NPWT in breast infections following breast implantation. It has been demonstrated that topical negative pressure used as closed incisional NPWT on closed operation wounds improves local blood circulation and reduces edema as well [18,19,20,21,22,23,24,25].

The incidence of PPBI is between 0.1 and 2.5% [26,27,28,29,30]. Because the overall incidence of PPBI is low, we present this pilot study of 12 patients who underwent implant removal and NPWTi-d due to PPBI. 

## 2. Materials and Methods

A retrospective analysis of 13 infected breasts in 12 female patients who underwent NPWTi-d due to peri-prosthetic breast infection was conducted. Due to the retrospective nature of this study, we did not recruit a control group of patients who received alternative treatment (PPBI without NPWTi-d). Cases were analyzed based on a complete medical record review, including regular patient follow-ups.

We used a computerized system to apply topical negative pressure with automatic instillation cycles (V.A.C. VeraFlo Therapy, Kinetic Concepts, Inc., San Antonio, TX, USA), using polyhexanide (0.4 mg/mL) (Lavasept, B. Braun Medical AG, Freiburg, Germany) as a rinsing solution. NPWTi-d was initiated immediately after removal of the implant. The first wound swab was taken directly after explantation of the implant and before irrigation of the pocket; a second wound swab was taken before wound closure. The instillation volume was adapted to the wound surface area so that the entire wound bed was covered with instillation fluid. Dwell frequency was 2-hourly. A dwell time of 20 min and pressure of −125 mmHg with continuous suction was set. Soaking time intervals were set between 2 and 3.5 h, depending on the clinical wound situation, and instillation of the wound was performed in every cycle. The foam was changed every 3–5 days with inspection of the wound bed to check for tissue granulation and signs of infection. 

In all cases, a complete capsulectomy was performed. In all but one case, the patients received antibiotics perioperatively, during NPWTi-d treatment and for another 5 days after wound closure/reconstruction/reimplantation. In the other case, antibiotic treatment was only performed as a single shot preoperatively before implant removal and for another 5 days after reconstruction. 

Data were analyzed for bacterial colonization of the implant pocket. Data from the first and last wound swabs before and after NPWT were collected. The wound swabs were obtained by the surgeon in a standardized manner by carefully passing the swab through the implant pocket. The number of different bacterial species (NDB) was counted, and the amount of bacteria (AB) in the culture was measured by the local Institute for Clinical Microbiology, according to recent studies [12,23]. Semiquantitative examination of the bacterial culture determined the extent of bacterial colonization of the implant pocket on an ordinal scale (1–4) for each bacteria (1: sparse, 2: moderate, 3: several, 4: plenty). Due to the heterogeneity of the bacterial colonization, the total amount of all bacteria found in the breast pocket was calculated by summing the semiquantitative ordinal scaled numbers of each bacteria. In addition, blood samples focused on inflammatory markers, including CRP and leukocyte count, were obtained before NPWTi-d and 5–6 days later during hospitalization.

Statistical analysis was performed using GraphPad Prism (Version 8.3.0 Prism 8, GraphPad Software Inc., La Jolla, CA, USA). The Wilcoxon signed-rank test was used for statistical analysis.

A *p*-value ≤ 0.05 was defined as a statistically significant (*) difference among the treatment groups. A *p*-value ≤ 0.001 was considered a highly significant (**) difference. Values are presented as mean ± standard deviation.

## 3. Results

### 3.1. Patient Demographics

We analyzed a total of 13 cases of PPBI in 12 female patients whose ages ranged from 24 to 94 years (median age: 52 years, mean age: 49.3 years). In 11 cases, the patients continued with regular follow-up at our outpatient clinic at 6 weeks and 6 months after the operation. In one case, follow-up ranged between 4 weeks and 5 years due to the retrospective nature of the study and the sometimes very long distance from the patient’s home to the hospital. In one case, there was no further consultation in our hospital records due to the patient’s advanced age of 94 years.

**Figure 1:**
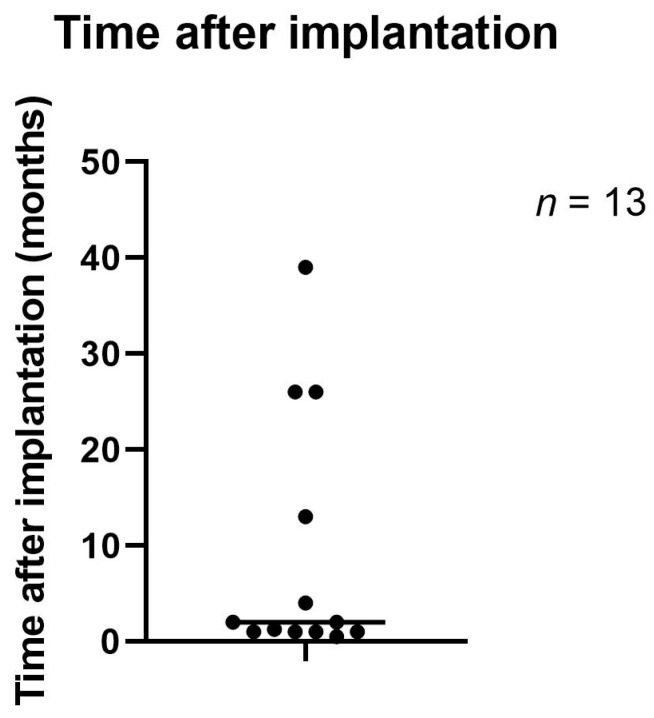
Time between implantation and explantation of the breast implant.

In 76.9% (*n* = 10) of the cases, the patients had received a silicone gel-filled implant for reconstructive reasons following cancer-related mastectomy, while 23.1% (*n* = 3) had received an implant for cosmetic reasons. Figure 1 shows the time range from implantation to removal of the implant due to PPBI. In one case, the time since the first implantation was unknown. The median time between implantation and explantation of the implant was 8 weeks, and the mean time in between was 36.2 weeks.

All patients had complete remission of their infection after NPWTi-d treatment following implant removal; the remission parameters were resolution of the clinical signs of infection (redness, swelling, pain), decreased serum CRP and serum leukocytes and absence of pus in the exudate. We performed secondary wound closures in 10 cases. One patient received a free muscle-sparing transverse rectus abdominis myocutaneous flap (ms-TRAM flap) and a free deep inferior epigastric artery perforator flap (DIEP flap) for reconstruction of both breasts in the later course, while another patient underwent repeated breast augmentation with a silicone implant. In three cases, split-thickness skin graft transplantation was necessary during the initial treatment due to the severity of the infection and related soft tissue loss. These patients received a free ms-TRAM flap or DIEP flap at a later stage.

In Table 1, patient characteristics are presented.

**Figure 2:**
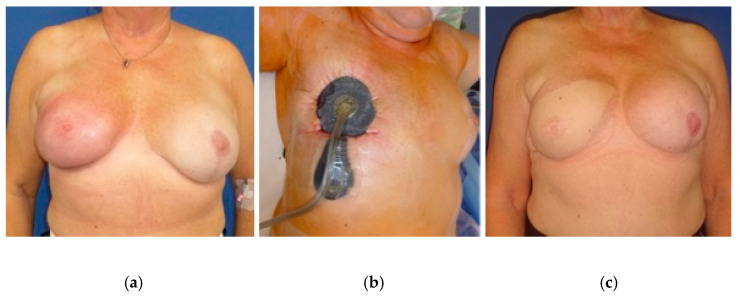
Example of NPWTi-d (**b**) in a 58-year-old patient with a history of breast cancer shown with PPBI. (**a**) Skin graft transplantation due to severe infection of the skin with necessary excision of the skin. (**c**) Five months later, the skin graft was removed, and the right breast underwent free ms1-TRAM flap reconstruction. The free ms1-TRAM flap was well perfused, and follow-up remained uneventful 6 weeks and 6 months later.

In Figure 2, an example of NPWTi-d in a 58-year-old patient with PPBI is shown. Figure 2a presents a severe infection of the skin with necessary excision and skin graft transplantation. In Figure 2b the topical treatment with NPWTi-d is illustrated. In Figure 2c a free ms1-TRAM flap is shown which was conducted five months later.

### 3.2. Bacterial Burden and Flora

**Figure 3:**
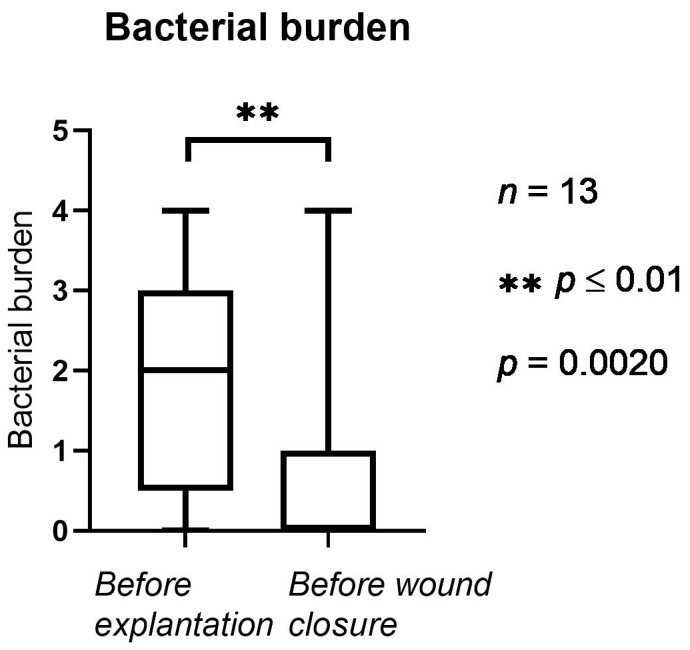
Bacterial burden in the implant pocket (1: sparse, 2: moderate, 3: several, 4: plenty). The horizontal lines mark the medians.

In Figure 3, the bacterial burden before and after NPWTi-d is presented. Before NPWTi-d, the bacterial burden counted an average of 1.92 (1.32 SD). Before wound closure (after NPWTi-d), the bacterial burden was statistically significantly lower, with an average of 0.76 (1.47 SD, *p* = 0.002). 

The average number of different types of bacteria before NPWTi-d was 0.92 (0.47 SD) and ranged from 0 to 2 different types of bacteria. In 7.7% (*n* = 1), two or more different types of bacteria were cultivated before NPWTi-d. In 76.9% (*n* = 10), there was one type of bacteria, and in 15.4% (*n* = 2), no bacteria were found. During the further course of treatment, all patients received antibiotics, including a cephalosporin and a antibiotic for anaerobic infections. Antibiotic therapy was adjusted according to the results of the wound swab cultures.

After NPWTi-d, the number of different types of bacteria decreased significantly, to an average of 0.23 (0.42 SD, *p* = 0.001). In the bacterial culture of the second wound swab, no cases showed two or more different bacteria. In 23.1% (*n* = 3) of the cases, one type of bacteria was found in the culture of the second wound swab, while in 76.9% (*n* = 10), no bacteria were found. In one case, the wound was colonized with multidrug-resistant bacteria (methicillin-resistant *Staphylococcus aureus*, MRSA) in the first wound swab. In the second wound swab (taken before wound closure), MRSA was no longer detected in the culture. The types of bacteria included MRSA, *Staphylococcus aureus*, *Staphylococcus epidermidis*, *Serratia marcescens*, *Staphylococcus warneri*, *Enterococcus faecalis*, *Proteus mirabilis* and *Bacillus* species. In 61.5% (*n* = 8) of the cases, swab analysis showed Gram-positive bacteria before NPWTi-d. No shift from Gram-positive to Gram-negative bacteria was observed. 

### 3.3. Serum CRP and Number of Leukocytes

**Figure 4:**
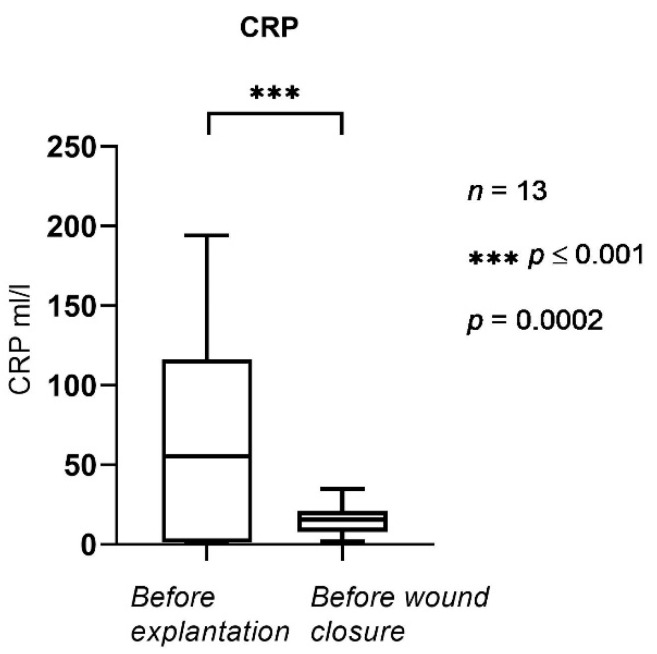
CRP levels before explantation and before wound closure. The horizontal lines mark the medians.

**Figure 5:**
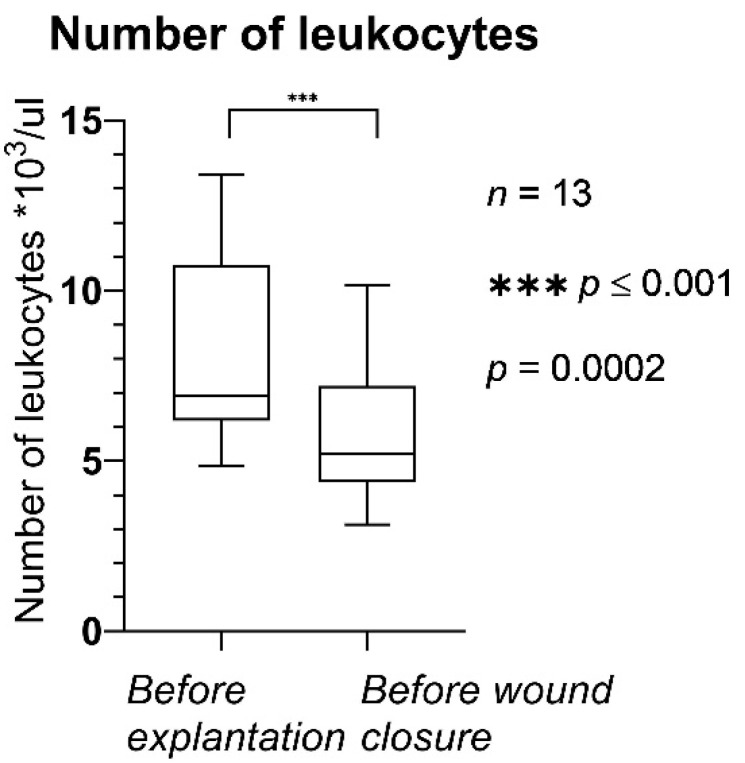
Number of leukocytes before explantation and before wound closure. The horizontal lines mark the medians.

In Figure 4 and Figure 5, CRP and the number of leukocytes in the serum are shown, respectively. 

Serum CRP (Figure 4) was statistically significantly lower before wound closure (after NPWTi-d) compared to the initial values obtained before removal of the implant. The mean was 55.3 mL/L before NPWTi-d and 15.5 mL/L before wound closure (*p* = 0.0002); serum CRP ranged from 1.8 to 34.8 mL/L before wound closure.

The number of leukocytes (Figure 5) was statistically significantly lower before wound closure (after NPWTi-d) compared to the values before the first operation. The mean values were 8.16 × 10^3^/µL before NPWTi-d and 5.83 × 10^3^/µL before wound closure (*p* = 0.0002); leukocyte counts ranged from 3.13 × 10^3^/µL to 10.17 × 10^3^/µL before wound closure.

The oldest patient (94 years) had undergone alloplastic breast augmentation for cosmetic reasons more than 10 years earlier. She presented to our emergency unit in poor general condition and in extreme pain with a fever. Physical examination showed a purulent wound with central skin necrosis and a unilateral exposed implant. Inflammatory markers were very high before NPWTi-d. The other implant was removed due to capsular fibrosis (grade IV) simultaneously with the secondary wound closure of the affected side. This patient had no further consultation at our hospital due to her advanced age. At the time of discharge from the clinic, her wounds were clinically inconspicuous.

### 3.4. Additional Treatment Data

**Figure 6:**
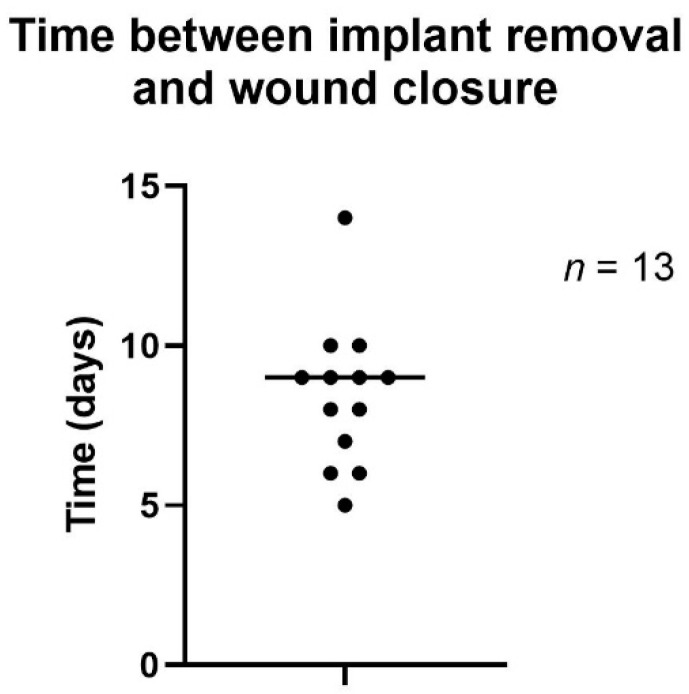
Time to wound closure after NPWTi-d.

Time to wound closure after NPWTi-d varied between 5 and 14 days, with a mean of 8.5 days (Figure 6).

**Figure 7:**
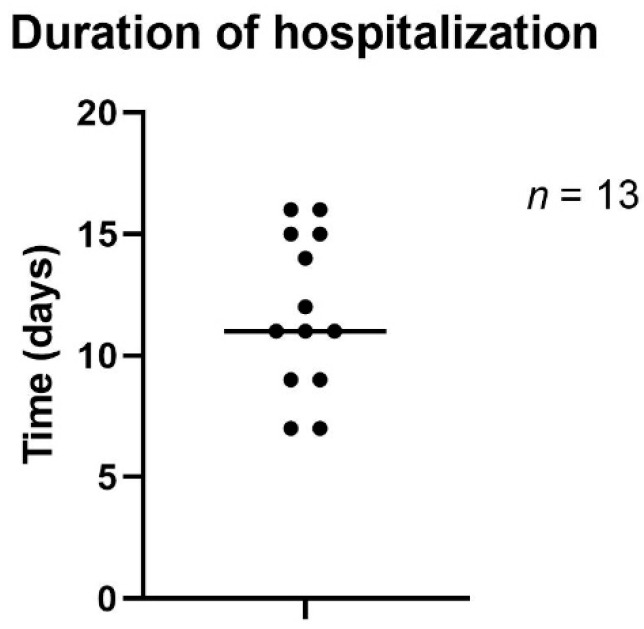
Duration of hospitalization.

Duration of hospitalization varied between 7 and 16 days, with a mean of 11.8 days (Figure 7).

## 4. Discussion

Peri-prosthetic breast infection is a severe complication that regularly results in multiple reoperations and risks an unsatisfactory outcome. In this context, the female breast is a unique anatomical wound location. Radiation therapy after breast cancer surgery is associated with elevated complications in terms of capsular fibrosis and surgical site infection [31,32]. Furthermore, it has been discussed that breast parenchyma is subject to bacterial colonization or contamination during breast implant procedures. Independent from the final coverage, which in irradiated breast wounds often includes a free flap to close the defect, technical advances, such as measuring the perfusion of the remaining tissue, may be necessary to ensure a successful outcome and minimize tissue loss [2,3,33,34].

Evaluating the options for PPBI treatment is difficult; data are either anecdotal or retrospectively collected because—as with other relatively infrequent surgical events—no prospective studies exist [35]. Some have called the management of severe infection or acute implant exposure following primary augmentation mammoplasty a classic surgical dilemma. According to Vasilakis et al., the inconsistency and paucity of published data preclude definitive conclusions regarding the optimal management of threatened implants [36]. However, despite insufficient published information about the effective management of these situations, Spear has recommended that device removal and delayed reinsertion is always a more conservative and predictable option, especially in seriously infected breasts or deficient soft tissue coverage when the implant does not appear to be salvageable [37]. A common treatment strategy after implant removal is reimplantation after weeks to months, but no consensus exists for standardized treatment in such cases.

Another issue is microperfusion of the skin and soft tissue in PPBI, which is essential for proper healing and hence the use of alloplastic material for reimplantation. There are currently several tools that can provide sufficient oxygenation and microperfusion to ensure adequate vascular supply to the affected tissue [2,3,18,19,33,38]. The use of noninvasive tools to validate tissue perfusion can be a helpful aid in optimizing reoperations.

In cases of PPBI, a major concern besides acute treatment is to create an ideal inflammatory-free anatomical site with maximum decontamination of the bacterial burden to facilitate reoperation and reduce the risk of further infectious sequelae. 

Besides implant removal and antibiotic therapy, which was applied to all patients in this study except one, the use of NPWTi-d decreased the bacterial burden in the former implant pocket. Moreover, the inflammatory blood markers decreased markedly over a short period of time (5–6 days) after NPWTi-d was applied. Even though implant removal and intravenous antibiotics are crucial in such cases, their actual impact on the bacterial burden has not been defined exactly. Additional intermittent instillation of the breast pocket seems to positively affect the bacterial burden in the wound bed. It must be mentioned that the true extent of NPWTi-d’s effect cannot be attributed exclusively to NPWTi-d without comparing its results to those of a control group. However, the reduction in bacterial load achieved through NPWTi-d reflects the results of previous studies that assessed the effects of NPWT/NPWTi-d on various types of contaminated wounds and different anatomical locations [12,35,39,40]. Especially for breast-associated interventions, such as reimplantation of an implant or autologous breast reconstruction, it is of the utmost importance to obtain clinically sterile wound conditions that are free of infection. Various complications, such as PPBI, capsular fibrosis and even breast implant-associated anaplastic large cell lymphoma, have been associated with bacterial colonization or bacterial biofilms. Within the first weeks and months after implantation, PPBI results primarily from Gram-positive bacteria, such as *Staphylococcus aureus* and *Streptococcus* [30,41]. In our study, the majority of cases (61.5%, *n* = 8) showed Gram-positive bacterial colonization. The most common bacterial species was *Staphylococcus epidermidis*, which affirms the possible transmission of bacteria from the skin into the wound [42]. As infections of the implant pocket with multidrug-resistant bacteria (e.g., MRSA) can cause severe complications due to limited antibiotic therapy regimen options, this study focused especially on the one patient who suffered from MRSA infection of the implant pocket. After NPWTi-d and targeted antibiotic therapy, MRSA was no longer detected in the implant pocket. In all cases, the infection resolved uneventfully so that successful wound closure could be achieved. 

This study demonstrated the positive effects of NPWTi-d on the healing process of peri-prosthetic wound infections and salvage of the breast implant pocket. In addition to its positive effects on bacterial burden and inflammation, NPWTi-d was able to prevent shrinkage in the former implant pocket and thereby preserve the skin envelope. Even though antibiotic use might influence bacterial analysis, it is of the utmost importance to decrease the bacterial burden in the former implant pocket, which was accomplished with NPWTi-d. The association between bacterial contamination or colonization and unfavorable sequelae following augmentation mammoplasty has been described extensively in the literature. Hence, in our opinion, NPWTi-d is a promising tool for treating PPBI, especially considering the goal of decreasing the bacterial burden as much as possible. This study presents an algorithm for problematic cases of infected and possibly exposed foreign material in the breast. Following removal of an infected breast implant, NPWTi-d using an antiseptic solution is recommended until local and systemic inflammatory markers have decreased and the local wound appears clinically clean. Between two and four changes of the NPWT dressing should be sufficient in most cases. If reimplantation with a silicone implant is planned, wound closure should be performed and reimplantation completed at the earliest 3 months after total healing of the infected breast and consecutive wounds. Planning autologous breast reconstruction offers the possibility of a direct approach following control of the local infection and treatment with NPWTi-d. This is possible because autologous breast reconstruction (e.g., using a DIEP flap) provides autologous tissue, including an independent microvascular supply. Furthermore, the patient’s own tissue has the capacity to overcome any possible residual bacterial colonization. Patients should be informed about the possibility of a second stage autologous breast reconstruction after an interval of several months. Consequently, this treatment algorithm might facilitate earlier breast reconstruction after PPBI, independent of the applied technique, leading to greater patient satisfaction and lower patient burden by shortening the period the patient has to live without a reconstructed breast.

Other authors have stated that NPWTi-d, which has become a game changer in the treatment of infected wounds, is an appropriate tool for the treatment of PPBI, and we agree [43,44]. However, it should be combined with intravenous antibiotic therapy perioperatively. The prolonged use of antibiotics in addition to NPWTi-d needs to be reconsidered after trials with more cases and according to the general condition of the patient, concomitant diseases and the severity of the infection. 

The findings of this study correspond to those of other publications [12,23,40,45,46]. Studies have found that, not only in PPBI but especially in chronic wounds, a significantly lower bacterial load was obtained after applying an antiseptic instillation solution as an adjunct to NPWT [12,47]. 

In all cases in this study, the breast implant pocket was preserved by NPWTi-d, which is consistent with the results of two studies published by Meybodi et al. in 2017 and 2021 [43,48]. 

Only a few previous cases have described the use of NPWT, with or without instillation, as a treatment option for peri-prosthetic infections [48,49,50]. Therefore, to confirm our findings, further studies with more patients are needed to develop a standardized therapy regimen or algorithm for using NPWTi-d to treat peri-prosthetic infections. As a study protocol that includes a prospective control group is not reasonable, comparing a greater number of patients with a historical patient group is recommended. Moreover, since inflammatory markers, such as CRP and leukocyte count, are not specific for any particular bacterial infection, it would be interesting to study whether indicators of systemic inflammation, such as laboratory markers, can be decreased more efficiently. This should be further investigated in the future. 

An already mentioned limitation of this study is that it lacked a control group, which prevented us from comparing the NPWTi-d data with other treatments. However, these data can descriptively show relatively fast remission of clinical infection parameters and shortened time to wound closure and overall duration of hospitalization [51].

Surgical debridement and antibiotics undoubtedly influence the treatment of infected wounds. Nevertheless, the results of this study confirm those of recent studies and underline the positive effect of NPWTi-d on bacterial burden.

## 5. Conclusions

Based on an analysis of 13 breast implant-related peri-prosthetic infections, the positive effects of NPWTi-d (which were already known from applications in complex and contaminated wounds in different anatomical locations) were confirmed in the unique context of breast infections. Based on these results, the use of NPWTi-d should be considered a valuable adjunct to the treatment of peri-prosthetic breast infections in addition to implant removal and antibiotic therapy. NPWTi-d could contribute to earlier second-stage breast reconstruction following an infection-free interval. However, further investigations with more cases are necessary to establish a treatment algorithm. 

## Figures and Tables

**Table 1:** Patient characteristics.

Patient No.	Age (In Years)	Reconstructive (R)/Cosmetic (C)	Infected Side	Radiation	Relevant Concomitant Diseases
1	52	R	Both	No	Hypertension, deep vein thrombosis of the lower leg
2	58	R	Left	Yes	None
2	60	R	Right	Yes	None
3	54	R	Right	No	Allergy to penicillin
4	40	R	Both	Yes	Allergy to novaminsulfone, tramadol and clindamycin
5	61	R	Right	No	BRCA2 mutation, hepatic steatosis, allergy to amoxicillin, clindamycin and tramadol
6	94	R	Left	Yes	Urinary incontinence, hypertension
7	24	C	Both	No	Smoker
8	41	R	Both	No	None
9	42	C	Right	No	None
10	55	R	Right	No	Pulmonary embolism
11	32	C	Both	No	None
12	46	R	Both	No	None
